# Rice Bran Supplements the Nutritional Density of Ready‐to‐Use Therapeutic Foods: A Targeted Nutrient and Non‐Targeted Metabolomic Analysis

**DOI:** 10.1002/fsn3.71448

**Published:** 2026-01-21

**Authors:** Annika M. Weber, Emma S. Bovaird, Sahar B. Toulabi, Silvia Barbazza, Moretta Damayanti Fauzi, Fildzah K. Putri, Khaerul Fadly, Kharisma Tamimi, Diva M. Calvimontes, Rimbawan Rimbawan, Zuraidah Nasution, Puspo Edi Giriwono, Frank T. Wieringa, Elizabeth P. Ryan

**Affiliations:** ^1^ Department of Food Science and Human Nutrition Colorado State University Fort Collins Colorado USA; ^2^ Department of Biochemistry and Molecular Biology Colorado State University Fort Collins Colorado USA; ^3^ Department of Horticultural Science Colorado State University Fort Collins Colorado USA; ^4^ Department of Health Sciences, Faculty of Science Vrije Universiteit Amsterdam Amsterdam the Netherlands; ^5^ UMR Qualisud, Université Montpellier, CIRAD, Institut Agro, IRD Université Avignon, Université de la Réunion Montpellier France; ^6^ Child Health Department of Mohammad Hoesin Hospital Palembang South Sumatera Indonesia; ^7^ Child Health Department of Faculty of Medicine Sriwijaya University Palembang South Sumatera Indonesia; ^8^ Savica Consultancy Surabaya Jawa Timur Indonesia; ^9^ Department of Community Nutrition IPB University Bogor Jawa Barat Indonesia; ^10^ South East Asia Food and Agriculture Science and Technology (SEAFAST) Center LRI PGK IPB University Bogor Indonesia; ^11^ Center for Human Development, Fundación Para la Salud Integral de los Guatemaltecos FUNSALUD Coatepeque Quetzaltenango Guatemala; ^12^ Department of Pediatrics, Center for Global Health University of Colorado Aurora Colorado USA; ^13^ Department of Food Science and Technology IPB University Bogor Jawa Barat Indonesia; ^14^ French National Research Institute for Sustainable Development (IRD) Montpellier France; ^15^ Department of Environmental and Radiological Health Sciences Colorado State University Fort Collins Colorado USA

## Abstract

The treatment of uncomplicated severe acute malnutrition includes ready‐to‐use therapeutic foods (RUTFs). Novel RUTF recipes aim for inclusion of locally sourced nutrient‐dense food ingredients for sustainability in product availability. This study investigated the incorporation of rice bran into RUTF formulations to enhance the bioactive nutrient profile. Experimental RUTFs were developed containing 0%, 5%, 7.5%, and 10% rice bran, without a vitamin/mineral premix for targeted nutrient and non‐targeted metabolite analysis. Additionally, an investigation was conducted analyzing the nutrient density and food safety of small‐scale mill‐sourced rice bran varieties collected from Guatemala and Cambodia for comparison to a US‐commercial rice bran. Targeted nutrient composition analysis of the RUTFs revealed dietary fiber, vitamin E, and vitamin B1 generally increased with higher rice bran content, though it was not dose dependent. The non‐targeted metabolite analysis identified 883 biochemicals across the four experimental RUTFs. Significant metabolite fold changes were identified for a variety of lipids, amino acids, carbohydrates, vitamins, and xenobiotics in 5%, 7.5%, and 10% rice bran‐RUTFs compared to the 0%. Analysis of small‐scale mill‐sourced rice brans from Guatemala and Cambodia showed variation in vitamin composition, with vitamin B3 averaging 37.1 mg/100 g and vitamin E ranging from 3.2 to 6.0 mg/100 g. These varieties also demonstrated variable microbial levels and trace metal contents, warranting continuous monitoring and evaluation in global supply chains. These findings support the feasibility of incorporating rice bran into RUTFs for malnutrition treatment and the benefit of screening locally sourced rice bran to address regional nutrient‐dense food product development and specifically for malnutrition treatment.

## Graphical abstract

Incorporating rice bran in ready‐to‐use therapeutic foods (RUTFs) was used for treatment of childhood malnutrition. RUTF nutrient analysis showed increased dietary fiber and vitamins E and B1. Non‐targeted metabolomics revealed significant changes in lipids, amino acids, carbohydrates, vitamins, and phytochemicals corresponding to rice bran. Rice bran from small‐scale mills in Guatemala and Cambodia had nutrient‐dense profiles and can be a locally sourced, novel food ingredient. These findings support global rice bran utility for childhood malnutrition treatment and prevention.

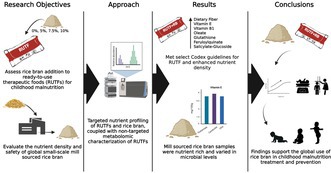

## 1 Introduction

Ready‐to‐use therapeutic foods (RUTFs) are nutrient‐dense, high‐calorie foods used in the treatment of uncomplicated severe acute malnutrition in children aged 6–59 months (FAO et al. 2023). The benefits of RUTFs include the ability to treat severe acute malnutrition in a community context, as many cases do not require hospitalization or are in rural areas where hospital access may not be feasible. Since the development and application of the commercial RUTF in the early 2000s, RUTFs have been composed primarily of peanuts, milk powder, sugar, vegetable oil, and vitamin/mineral premix. The most recent requirements for RUTF macro‐ and micronutrient content are outlined in the Codex Alimentarius (FAO et al. 2023). However, attention has been placed on alternative RUTF formulations, focusing on in‐country production, locally sourced ingredients, and culturally appropriate taste, flavors, and texture preferences (Fetriyuna et al. 2023; Pajak et al. 2025). A study by Rachmadewi et al. in 2023 found that alternative RUTFs that used ingredients readily available in Indonesia, such as mung bean, soybean, or peanut, were as effective in mean weight gain and were better accepted than a standard, imported RUTF (Rachmadewi et al. 2023). The use of local ingredients could mitigate reliance on expensive, imported ingredients (e.g., milk powder, vitamin/mineral premix), overall decreasing production costs and increasing accessibility. In addition, malnutrition treatment should not be delayed by disruptions in the global supply chains due to conflicts and geopolitical instability.

Despite increased interest in alternative RUTF formulations, many malnutrition treatment programs rely on standardized RUTF formulations with imported ingredients. The in‐country production of RUTFs is also constrained by insufficient data on nutritional quality, safety, and functional properties of locally available ingredients. Some progress exists in Indonesia for protein and lipid content analysis of taro, groundnut, and mung bean flours intended for use in local malnutrition treatment (Fetriyuna et al. 2021), yet comprehensive nutritional composition and food safety evaluations remain limited. Locally sourced chickpea and green banana‐based RUTFs were assessed in Bangladesh (Mostafa et al. 2022, 2024), lentil‐ and sorghum‐based products in Malawi and Uganda (Bahwere et al. 2017; Walsh et al. 2023), and fish‐based or mung bean‐enriched formulations in Pakistan and Cambodia (Javed et al. 2021; Sigh et al. 2018). While these examples demonstrate feasibility, they highlight the global need for comparative and standardized analyses in each region.

Rice bran, the outer layer of brown rice and a co‐product of rice milling, is a nutrient‐dense food ingredient for examination in the context of RUTF development. This underutilized food ingredient is rich in a variety of lipids, amino acids, and prebiotic fibers, as well as vitamins, minerals, and assorted phytochemicals (Borresen and Ryan 2014; Kinyuru et al. 2015; Zarei et al. 2017, 2018). Rice bran has also demonstrated functional properties to protect against diarrheal diseases via inhibition of pathogen entry and replication (Goodyear et al. 2015; Kumar et al. 2012), enhanced mucosal immunity (Lei et al. 2016; Yang et al. 2014), and gut barrier function (Goodyear et al. 2015; Vilander et al. 2022). Infants at high risk for malnutrition in Mali and Nicaragua were supplemented daily with rice bran for 6 months and showed improved linear growth while increasing gut microbiome diversity (Vilander et al. 2022; Zambrana et al. 2019). Rice bran is available in many countries (Tan et al. 2023), and there is tremendous potential for expanding utility with affordable reincorporation into human food supply chains (Asian Development Bank 1989; Das et al. 2024; Gul et al. 2025). A comprehensive examination of heavy metal risks associated with rice production is essential to ensure food safety of the rice bran (Gu et al. 2018; Weber et al. 2021). Prior to our study, a thorough examination of the nutritional contributions and safety of rice bran in the context of malnutrition treatment foods has been limited (Kinyuru et al. 2015).

The Solutions to Enhance Health with Alternative Treatments (SEHAT) clinical trial in Indonesia established the first assessment of rice bran as an ingredient in RUTFs in a double blinded randomized controlled trial (Weber et al. 2023). The SEHAT trial measured the feasibility of incorporating rice bran into RUTFs and for the gut microbiome‐targeted treatment of malnutrition. This study of experimental RUTFs and locally sourced rice bran from countries experiencing malnutrition complements the SEHAT trial outcomes by providing nutrient density and non‐targeted metabolite profile analyses of rice bran and rice bran‐RUTFs. Notably, these experimental RUTFs were identical in formulation, yet prepared without the vitamin/mineral premix. This approach allows evaluation of the direct contribution of rice bran to the nutritional composition of RUTFs. The nutrient composition and safety testing of rice bran collected from small‐scale mills in Guatemala and Cambodia showed that systematic field testing for nutrient profiles and safety of rice bran is possible and provides evidence for locally sourcing rice bran to combat malnutrition while addressing global food security.

## 2 Materials and Methods

### 2.1 Experimental Ready‐To‐Use‐Therapeutic Food (RUTF) Development

The RUTFs were produced by the Southeast Asia Food and Agricultural Science and Technology (SEAFAST) Centre and the Department of Community Nutrition, Bogor Agricultural University in Bogor, Indonesia. The RUTF recipes used the same basic composition as the Bregas Roll, which has been previously documented (Rachmadewi et al. 2023; Rimbawan et al. 2024; Weber et al. 2023), but did not contain the typical vitamin/mineral premix added to RUTFs in order to assess the dose‐dependent nutrient contribution of rice bran. All manufacturing processes were the same as previously described, with minor adjustments to account for the different amounts of rice bran added to the formula and without the addition of the vitamin/mineral premix. The RUTFs consist of a wafer filled with a paste composed of palm oil, milk powder, skim milk powder, whey protein concentrate, sugar, peanut butter, wheat flour, rice flour, maltodextrin, and flavors of either vanilla or cocoa powder. The experimental RUTFs had either 0% rice bran added, and for the RUTFs with 5%, 7.5%, or 10% rice bran, maltodextrin was reduced, and small modifications were made to skim milk powder, sugar, and wheat flour quantities (Table 1). All 0%, 5%, 7.5%, and 10% rice bran RUTFs were made in both chocolate and vanilla flavors. Given the substantial quantity of rice bran required to produce these RUTFs and to ensure consistency, a heat‐stabilized commercial‐US rice bran was supplied by Colorado State University (SN100, purchased from Stabil Nutrition LLC, St Louis, MO, USA). All other ingredients (except whole milk powder, skim milk powder and whey protein concentrate) were sourced locally, and in accordance with Indonesian food quality and safety standards. The RUTFs were stored in a standard production facility storage unit maintained at 30°C ± 3°C and 60% relative humidity until subsequent shipment to Colorado State University and stored at 4°C until analysis. All RUTFs that were experimental samples tested here were used solely for nutritional composition analysis.

**TABLE 1:** Experimental RUTF recipe and ingredient profiles with increasing amounts of rice bran.

Ingredients, g/100 g	0% rice bran‐RUTF	5% rice bran‐RUTF	7.5% rice bran‐RUTF	10% rice bran‐RUTF
Palm oil	21.70	20.80	20.6	20.0
Whole milk powder	17.60	18.10	18.1	18.1
Peanut butter	8.60	8.60	8.60	8.60
Sugar	16.30	14.40	14.4	13.0
Whey protein concentrate	7.90	8.00	8.00	8.00
Skim milk powder	6.70	5.80	4.00	4.00
Wheat flour	9.20	8.70	8.30	7.60
Rice flour	9.40	8.40	8.00	8.00
Maltodextrin	0.70	0.30	0.30	0.50
Rice bran	0.00	5.00	7.50	10.0
Cocoa powder/vanilla	1.40	1.40	1.40	1.40
Proprietary ingredients (binder)	0.50	0.50	0.80	0.80

### 2.2 Quantified Nutrient Composition of Experimental RUTFs

To prepare the RUTFs for the nutrient analysis, 15 chocolate and 15 vanilla RUTF wafer rolls of the 0% rice bran‐RUTF were homogenized in a high‐speed food processor to create the 0% rice bran‐RUTF batch for testing (Figure S1). The same procedure was performed for the 5% rice bran‐RUTF, 7.5% rice bran‐RUTF, and 10% rice bran‐RUTF respectively, and were considered representative batches. 400 g of each representative batch was sent to IEH‐Warren Laboratory, Greeley, Colorado, for quantified nutrient composition analysis in single measurements. IEH‐Warren Laboratory is an accredited nutritional analysis laboratory which uses established analytical methods. Targeted nutrients included: Fat—Acid Hydrolysis (Fat—Acid Hydrolysis AOAC 945.44), Protein (AOAC 990.03/992.23/992.15 (LECO)), Carbohydrate (Carbohydrate Calculation), Calories (Calories Calculation), Ash (Ash AOAC 920.153 standard), Moisture (Moisture AOAC 950.46), Dietary Fiber (Dietary Fiber AOAC 991.43), Insoluble Fiber (Insoluble Fiber AOAC 991.43), Soluble Fiber (Soluble Fiber AOAC 991.43), Vitamin E (Vitamin E alpha‐tocopherol AACC‐86.06 Modified), Vitamin B6 Pyridoxine Hydrochloride (Vitamin B6 Pyridoxine Hydrochloride Pyridoxine AOAC 2004.07), Vitamin B3 (Niacin, Niacinamide AOAC‐944.13), Vitamin A (Total) (Vitamin A (Total) WRE 054), Vitamin B1 (Thiamine (B1) LC–MS/MS), Vitamin B2 (Riboflavin (Vitamin B2) JAOAC Vol 76, No 5, 1156, 1993), and Peroxide (Peroxide AOCS Cd 8–53).

### 2.3 Metabolomic Analysis of Experimental RUTFs

#### 2.3.1 Sample Preparation

Fifty milligram aliquots were taken in triplicate from each representative batch of RUTF, as described above (0% rice bran‐RUTF [*n* = 3], 5% rice bran‐RUTF [*n* = 3], 7.5% rice bran‐RUTF [*n* = 3], and 10% rice bran‐RUTF [*n* = 3]) for metabolite analysis and analyzed separately (Figure S1). Samples were sent to Metabolon Inc. (Morrisville, NC) for non‐targeted metabolite profiling using ultrahigh‐performance liquid chromatography‐tandem mass spectroscopy (UPLC‐MS/MS). Before processing, all samples were held at −80°C. Samples were prepared using the automated MicroLab STAR system from Hamilton Company. Several recovery standards were added prior to the first step in the extraction process for Quality Control (QC). To remove protein, dissociate small molecules bound to protein or trapped in the precipitated protein matrix, and to recover chemically diverse metabolites, proteins were precipitated with methanol under vigorous shaking for 2 min (Glen Mills GenoGrinder 2000) followed by centrifugation (spun at 1500 g). Each sample was then divided into aliquots for ultra‐high performance liquid chromatography tandem mass spectrometry (UPLC‐MS/MS) analysis. Several types of controls were included with the experimental samples. The pooled matrix sample was generated by taking a small volume of each experimental sample and served as a technical replicate throughout the data set. Extracted water samples served as process blanks, and a cocktail of QC standards was selected to avoid interference with the measurement of endogenous nutrients/compounds. The spike of QC standards into every sample supported instrument performance monitoring and aided chromatographic alignment prior to analysis.

#### 2.3.2 Ultra‐High Performance Liquid Chromatography Tandem Mass Spectrometry (UPLC‐MS/MS)

Aliquots underwent reverse‐phase UPLC‐MS/MS with positive electrospray ionization (ESI), reverse‐phase UPLC‐MS/MS with negative ESI, and hydrophilic interaction liquid chromatography tandem mass spectrometry (HILIC/UPLC‐MS/MS) with negative ESI. All methods utilized a Waters ACQUITY ultra‐performance liquid chromatography (UPLC) and a Thermo Scientific Q‐Exactive high resolution/accurate mass spectrometer interfaced with a heated electrospray ionization (HESI‐II) source and Orbitrap mass analyzer operated at 35,000 mass resolution. The dried sample extracts were then reconstituted in solvents compatible to each of the four methods. Each reconstitution solvent contained a series of standards at fixed concentrations to ensure injection and chromatographic consistency. One aliquot was analyzed using acidic positive ion conditions, optimized for hydrophilic compounds. Here, the extract was gradient eluted from a C18 column (Waters UPLC BEH C18‐2.1 × 100 mm, 1.7 μm) using water and methanol, containing 0.05% v/v perfluoropentanoic acid (PFPA) and 0.1% v/v formic acid (FA). Another aliquot was also analyzed using acidic positive ion conditions. Here, the extract was gradient eluted from the same C18 column using methanol, acetonitrile, water, 0.05% PFPA and 0.01% FA and was operated at an overall higher organic content. Another aliquot was analyzed using basic negative ion optimized conditions using a separate dedicated C18 column. The basic extracts were gradient eluted from the column using methanol and water, with 6.5 mM Ammonium Bicarbonate at pH 8. The last aliquot was analyzed via negative ionization following elution from a HILIC column (Waters UPLC BEH Amide 2.1 × 150 mm, 1.7 μm) using a gradient consisting of water and acetonitrile with 10 mM Ammonium Formate, pH 10.8 (HILIC). The MS analysis alternated between MS and data‐dependent MS^n^ scans using dynamic exclusion. The scan range varied by method and covered 70–1000 m/z.

#### 2.3.3 Data Extraction and Compound Identification

Data extraction was performed and processed by Metabolon Inc. Mass spectral peaks were quantified and identified by comparison to library entries of ~5400 purified standards and recurrent unknown entities. Biochemical identification was based on retention index (RI) within a narrow RI window of the proposed identification, accurate mass match to the library ±10 ppm, and the MS/MS forward and reverse scores between the experimental data and authentic standards. The MS/MS scores are based on a comparison of the ions present in the experimental spectrum to the ions present in the library spectrum. Raw abundances were determined using quantified area under the curve for each peak.

### 2.4 Mill‐Sourced Rice Bran Nutrient Analysis

Rice bran samples were collected from small‐scale mills in Guatemala and Cambodia to represent locally available sources of rice bran for development as human food ingredients useful for malnutrition treatment formulations. Freshly milled bran from common local rice varieties in each country was identified in collaboration with rice miller input. The local Guatemala and Cambodia rice mills represented rice processing systems in rural communities with childhood malnutrition. These samples represent real‐world rice agriculture‐food system variability in rice bran nutrient content and heavy metal accumulation for practical translation of rice bran food ingredient production systems in diverse low‐middle income country (LMIC) settings.

The two Guatemalan rice bran samples (Guatemala I and Guatemala II) were collected from local mills in southwest Guatemala. Guatemala I was collected in December 2019, stored at 4°C for 1 month, and was then heat‐stabilized in an oven at 100°C for 5 min in Fort Collins, Colorado. Guatemala II was collected in March 2020 and was heat‐stabilized in an oven at 100°C for 5 min within 24 h of mill collection in Trifinio, Guatemala. Cambodian rice bran samples were collected in 2019 in the Kampong Chhnang province and heat stabilized within 24 h at the Ministry of Agriculture, Forestry and Fisheries post‐harvest research department in Phnom Penh. All rice brans were shipped to Fort Collins, Colorado at room temperature. Quantified nutrient compositions of the rice brans were analyzed in a single measurement at IEH‐Warren Laboratory, Greeley, Colorado. The targeted nutrients included: Vitamin B1 (Thiamine JAOAC Vol 76, No 5, 1156, 1993), Vitamin B2 (Riboflavin JAOAC Vol 76, No 5, 1156, 1993), Vitamin B3 (Niacin, Niacinamide AOAC‐944.13), Vitamin B5 (Pantothenic Acid—B5 METHOD AOAC‐960‐46), Vitamin B6 (Pyridoxine Hydrochloride AOAC 2004.07), Vitamin B9 (Folic Acid METHOD DFE3 AOAC‐944.12), Vitamin E (alpha‐tocopherol AACC‐86.06 Modified). A heat‐stabilized commercial‐US rice bran (SN100) was purchased from Stabil Nutrition LLC (St Louis, MO, USA) for product comparison, and the rice bran nutrient profile is provided in Table S1.

Microbiological evaluation by IEH‐Warren Laboratory, Greeley, Colorado, includes *Salmonella* spp. (MB217.07; AOAC Cert# 100701, PCR), *Escherichia coli* ( *E. coli* ) count (MB072.03; CMMEF, 4th Ed., Ch. 8, Spread Plate), Yeast and Mold count (MB074.01; FDA BAM, Ch.18, Plate), and Total Enteric Count (M219.03, CMMEF, 4th Ed., Ch. 8, Spread Plate). Heavy metal evaluation includes enumeration of lead (EPA 6020 modified), cadmium (EPA 6020 modified), and arsenic (EPA 6020 modified) by IEH‐Warren Laboratory, Greeley, Colorado. Rice bran from Cambodia Mill II was not tested due to lack of resources. The SN100 rice bran food safety profile was provided by Stabil Nutrition.

### 2.5 Statistical Analysis

Data from the targeted nutritional composition of the RUTFs were analyzed in R (v4.4.0) and GraphPad Prism (version 10.2.3) for descriptive analysis. Non‐targeted metabolite raw abundances were batch normalized, where for each metabolite, the raw values in the experimental samples were divided by the median of those samples in each instrument batch, giving each batch and, thus, the metabolite a median of one. The minimum value across all batches in the median scaled data for each metabolite was imputed for the missing values. Batch‐imputed median‐scaled relative abundances were used for descriptive comparisons. The batch‐imputed data was then transformed using the natural log. The average of each RUTF log‐transformed data was used for data analysis (0% rice bran‐RUTF [*n* = 3], 5% rice bran‐RUTF [*n* = 3], 7.5% rice bran‐RUTF [*n* = 3], and 10% rice bran‐RUTF [*n* = 3]). Variable important in the project (VIP) scores were determined from partial least squares discriminant analysis (PLS‐DA) Important Features tool in MetaboAnalyst (v6.0) online analysis software, after Pareto Scaling. Metabolites with a VIP score > 1.0 were considered contributors to discrimination potential between samples. Welch's two‐sample *t*‐tests were used to identify biochemicals that differed significantly between experimental groups, and the fold differences of the rice bran‐RUTFs compared to 0% rice bran‐RUTF. To account for false discovery rate errors, a *q*‐value was calculated for each metabolite. Statistical significance was determined using *p* < 0.05, and a *q*‐value of *q* < 0.10 indicated a high‐confidence result.

## 3 Results and Discussion

### 3.1 Experimental RUTF Formulation and Targeted Nutrient Composition

The ingredient formulations for the four RUTFs (without vitamin/mineral premix) are shown in Table 1 for 0% rice bran‐RUTF, 5% rice bran‐RUTF, 7.5% rice bran‐RUTF, and 10% rice bran‐RUTF. To accommodate the increasing percentage of rice bran, maltodextrin (corn soluble fiber) was decreased, and small modifications were made to the amounts of skim milk powder, sugar, and wheat flour.

The Codex Alimentarius Guidelines provide international standards for the safety and nutritional composition of RUTF formulations (FAO et al. 2023). To assess the effect of rice bran on the RUTF nutritional profile, the concentrations of macronutrients and select micronutrients in RUTFs (without vitamin/mineral premix) were examined to determine the percent fulfillment of these guidelines (Table 2). Overall, the nutrient density changes with increasing percentages of rice bran, though the absolute nutrient quantifications were not all proportionally dose dependent on rice bran. All RUTFs met Codex guidelines for energy (520 to 550 kcal/100 g), whereby calories in the RUTFs ranged from 528 to 555 kcal per 100 g. All RUTFs satisfied the minimum lipid Codex guideline of 26 g/100 g and ranged from 30.1 to 35.4 g/100 g. The greatest magnitude difference in lipids was a 5.3 g increase between the 0% rice bran‐RUTF and the 7.5% rice bran‐RUTF. The lipid concentration can be attributed to the increasing levels of rice bran. Rice bran is rich in lipids (20% lipids) as it contains mono and polyunsaturated fatty acids (palmitic acid and linoleic acid), gamma‐oryzanol, tocopherols/tocotrienols, and phytosterols (Gul et al. 2025). Many rice bran lipids have exhibited cholesterol‐lowering, antioxidant, and even gut microbiome modulation properties in animal studies and people (Barros Santos et al. 2023; Castanho et al. 2019; Park et al. 2025).

**TABLE 2:** RUTF nutrient content and percent fulfillment of Codex guidelines with inclusion of rice bran^a^.

Nutrient, g/100 g	0% rice bran‐RUTF	Fulfillment (%)^a^	5% rice bran‐RUTF	Fulfillment (%)^a^	7.5% rice bran‐RUTF	Fulfillment (%)^a^	10% rice bran‐RUTF	Fulfillment (%)^a^
Energy (kcal/100 g)	528	101.54	544	104.62	555	106.73	548	105.38
Moisture^b^	3.23	—	3.03	—	2.71	—	2.56	—
Ash^b^	2.32	—	3.20	—	2.81	—	2.94	—
Lipid	30.1	115.73	33.8	129.92	35.4	136.27	33.91	130.42
Protein	13.3	102.08	14.2	109.31	9.30	71.54	13.59	104.54
Carbohydrates^b^	51.0	—	46.0	—	49.8	—	47.0	—
Dietary Fiber^b^	0.80	—	2.20	—	4.50	—	4.60	—
Soluble Fiber^b^	< 0.5	—	1.60	—	2.00	—	2.20	—
Insoluble Fiber^b^	0.80	—	0.60	—	2.50	—	2.40	—
Vitamin E (mg/100 g)	21.1	105.50	21.6	108.00	21.6	108.00	44.6	223.00
Vitamin B1 (mg/100 g)	0.14	28.00	0.35	69.80	0.35	71.00	0.38	76.00
Vitamin B2 (mg/100 g)	0.22	13.75	0.22	13.75	0.30	18.75	0.13	8.00
Vitamin B3 (mg/100 g)	6.00	120.00	5.90	118.00	5.82	116.40	13.81	276.20
Vitamin B6 (mg/100 g)	0.01	1.67	0.02	3.00	0.02	2.67	0.16	26.67
Vitamin A (Total) (mg/100 g)	0.29	35.66	0.27	34.35	0.18	22.15	1.1	162.50
Peroxides (meq/kg)^b^	16.4	—	16.2	—	9.30	—	38.9	—

^a^ Fulfillment based on guidelines for certain nutrients defined in Codex Alimentarius 2022, typical RUTF vitamin/mineral premix was omitted.

^b^ Not defined in Codex Alimentarius guidelines.

According to Codex guidelines, RUTFs should contain at least 2.5 g of protein per 100 kCal, which translates to 13 g of protein per 100 g. Here, the protein content in the RUTFs ranged from 9.30 to 14.20 g/100 g. The 0%, 5%, and 10% rice bran‐RUTFs met the Codex protein guidelines for protein. Rice bran has a protein content of 12%–15%, providing amino acids including tyrosine and essential amino acids, such as tryptophan, lysine, leucine, and isoleucine, many of which have antioxidant properties and can contribute to cognitive development (Wen et al. 2025; Zaky et al. 2020; Zarei et al. 2017). The failure to reach Codex requirements for the 7.5% rice bran‐RUTF is likely due to recipe modifications, especially the changes to overall skim milk powder quantity with the intent to maintain texture quality. The combination of reduction in skim milk powder and 7.5% rice bran resulted in this lower than standard protein content, which also showed lower stability and homogeneity, which was not observed or corrected in the 10% rice bran RUTF formula, due to increased maltodextrin for stability and homogeneity, and additional protein content found in rice bran (Table 1).

Although standards for RUTF development require that carbohydrates should provide any remaining energy needs, neither the total carbohydrates nor total fiber are specified in the current 2023 CODEX guidelines (FAO et al. 2023). Total carbohydrates ranged from 46.0 to 51.0 g/100 g, while total dietary fiber content in the RUTFs increased with rice bran inclusion, from 0.80 g/100 g in the 0% rice bran‐RUTF to 4.60 g/100 g in the 10% rice bran‐RUTFs. A similar trend was observed for soluble fiber and insoluble fiber that increased incrementally, though it was not dose‐dependent. Rice bran total fiber comprises 80% insoluble fiber including cellulose, hemicellulose, and arabinoxylans (Kulathunga et al. 2023; Manzoor et al. 2023), as well as soluble fiber (20%) such as pectin and beta‐glucans (Cho and Samuel 2009; Zhao et al. 2018). Therefore, rice bran has the capacity to enhance total dietary fiber of finished food products with delivery of prebiotics that can support gut microbiota metabolism and gut mucosal barrier health (Henderson et al. 2012; Komiyama et al. 2011; Yang et al. 2015).

Without the vitamin/mineral premix included in the experimental RUTFs, this targeted analysis of selected vitamins revealed that rice bran‐RUTFs achieve Codex guidelines for certain vitamins. All RUTFs fulfilled the vitamin E requirements in the Codex guidelines of > 20 mg/100 g, containing relatively similar quantities across 0%, 5%, and 7.5% rice bran‐RUTFs, ranging from 21.1 to 21.6 mg/100 g, and was 44.6 mg/100 g in 10% rice bran‐RUTF. Vitamin B1 ranged from 0.14 to 0.38 mg/100 g between the experimental RUTFs. None of the experimental RUTFs met the Codex guidelines for vitamin B1 of > 0.5 mg/100 g. However, vitamin B1 did increase with higher percentages of rice bran inclusion and reached ~76% fulfillment of Codex guidelines in the 10% rice bran‐RUTF. Vitamin B2 ranged from 0.13 to 0.3 mg/100 g, with the Codex guideline at 1.6 mg/100 g. All RUTFs fulfilled vitamin B3 guidelines of > 5 mg/100 g, and were consistent between 0%, 5%, and 7.5% rice bran‐RUTFs, ranging from 5.82 to 6.00 mg/100 g. The 10% rice bran‐RUTF was the highest in vitamin B3 at 13.81 mg/100 g. This is likely due to the whey protein, cereals, and rice bran in the RUTF. Vitamin B6 was relatively similar among the RUTFs, ranging from 0.01 to 0.16 mg/100 g, though none of the RUTFs met the Codex guidelines of > 0.6 mg/100 g. The 0%, 5%, and 7.5% rice bran‐RUTFs did not meet total vitamin A Codex guidelines of 0.8 to 1.6 mg/100 g. However, 10% rice bran‐RUTF had a higher total vitamin A concentration (1.1 mg/100 g), thereby meeting the Codex guideline range. While rice bran is not typically rich in vitamin A, it does contain a small amount of carotenoids that may convert to vitamin A (Belefant‐Miller and Grunden 2014; Nagendra Prasad et al. 2024). However, this measurement should be verified by additional analyses and interpreted with caution to ensure consistent vitamin A concentration results.

Peroxide levels ranged from 9.30 to 38.9 meq/kg. This range indicates moderate to high peroxide levels and this oxidation of lipids was expected from storage conditions with possible heat exposure in Bogor, West Java, Indonesia and during shipment to Colorado, USA prior to analysis. These levels are not considered to pose direct food safety risks as rancidity thresholds are typically set at 40 meq/kg (Food Safety and Standards Authority of India 2016).

In summary, increasing rice bran inclusion in RUTFs enhanced lipid, fiber, and selected micronutrients (vitamin E, B1, B3, and A) while maintaining compliance with Codex standards. This report demonstrates the strong potential for rice bran to improve RUTF nutritional quality and functional properties prior to the addition of a micronutrient premix.

### 3.2 Non‐Targeted Metabolomic Analysis of Experimental RUTFs

**FIGURE 1:**
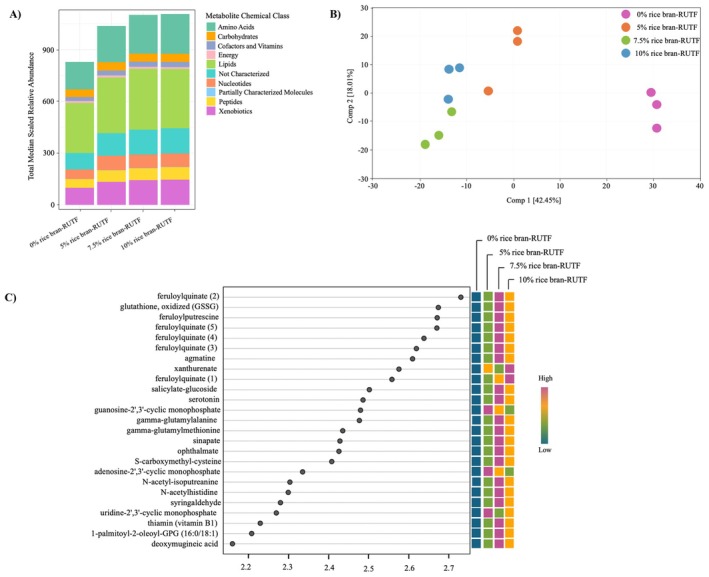
Non‐targeted metabolomic analysis of the experimental RUTFs. (A) Total of median scaled relative abundance metabolites within chemical classes for each RUTF (*n* = 3) with different doses of rice bran. (B) Principal component analysis (PCA) of metabolites in 0% rice bran‐RUTF (*n* = 3), 5% rice bran‐RUTF (*n* = 3), 7.5% rice bran‐RUTF (*n* = 3), and 10% rice bran‐RUTF (*n* = 3). (C) Top‐ranked 25 variable importance in projection (VIP) scores based on partial least squares‐discriminant analysis (PLS‐DA). VIP score reflects a measure for metabolite impact as a discriminant feature among the RUTFs.

A total of 883 detected metabolites, including 785 with identity annotations, were in the non‐targeted metabolomic analysis across all four experimental RUTFs. The major chemical classes of annotated compounds consisted of Amino Acids, Carbohydrates, Cofactors, and Vitamins, Energy Metabolism, Lipids, Nucleotides, Peptides, and Xenobiotics. Median scaled relative abundance sums for each chemical class were calculated across the RUTFs (Figure 1A). Lipid compounds accounted for the greatest number of median scaled relative abundances in all RUTFs, followed by Amino Acids and Xenobiotics. In general, each chemical class median scaled relative abundance increased with higher percentages of rice bran inclusion, indicating increases in nutrient and phytochemical density of the rice bran‐RUTFs.

The PCA analysis in Figure 1B revealed a distinct separation between the RUTFs containing rice bran (5%, 7.5%, and 10% rice bran‐RUTF) and the 0% rice bran‐RUTF. This separation is supported by 42.45% of the variance explained by Principal Component 1 (PC1) and 18% by Principal Component 2 (PC2), indicating a strong differentiation in their metabolomic profiles and highlighting the influence of rice bran inclusion on the overall metabolomic composition of the RUTFs.

VIP scores were calculated from the PLS‐DA model to identify influential metabolites that distinguish RUTF groups. Table S2 contains the list of metabolites with VIP score > 1.0 and are considered significant contributors to each dose‐RUTF group separation, further highlighting the observed metabolic profile differences. The 25 metabolites with the highest VIP scores are depicted in Figure 1C. The metabolites with the highest VIP scores were generally within the chemical classification of Xenobiotics, such as many of the feruloylquinate metabolites which are plant‐derived phytochemicals or were within the chemical class of Amino Acids.

**FIGURE 2:**
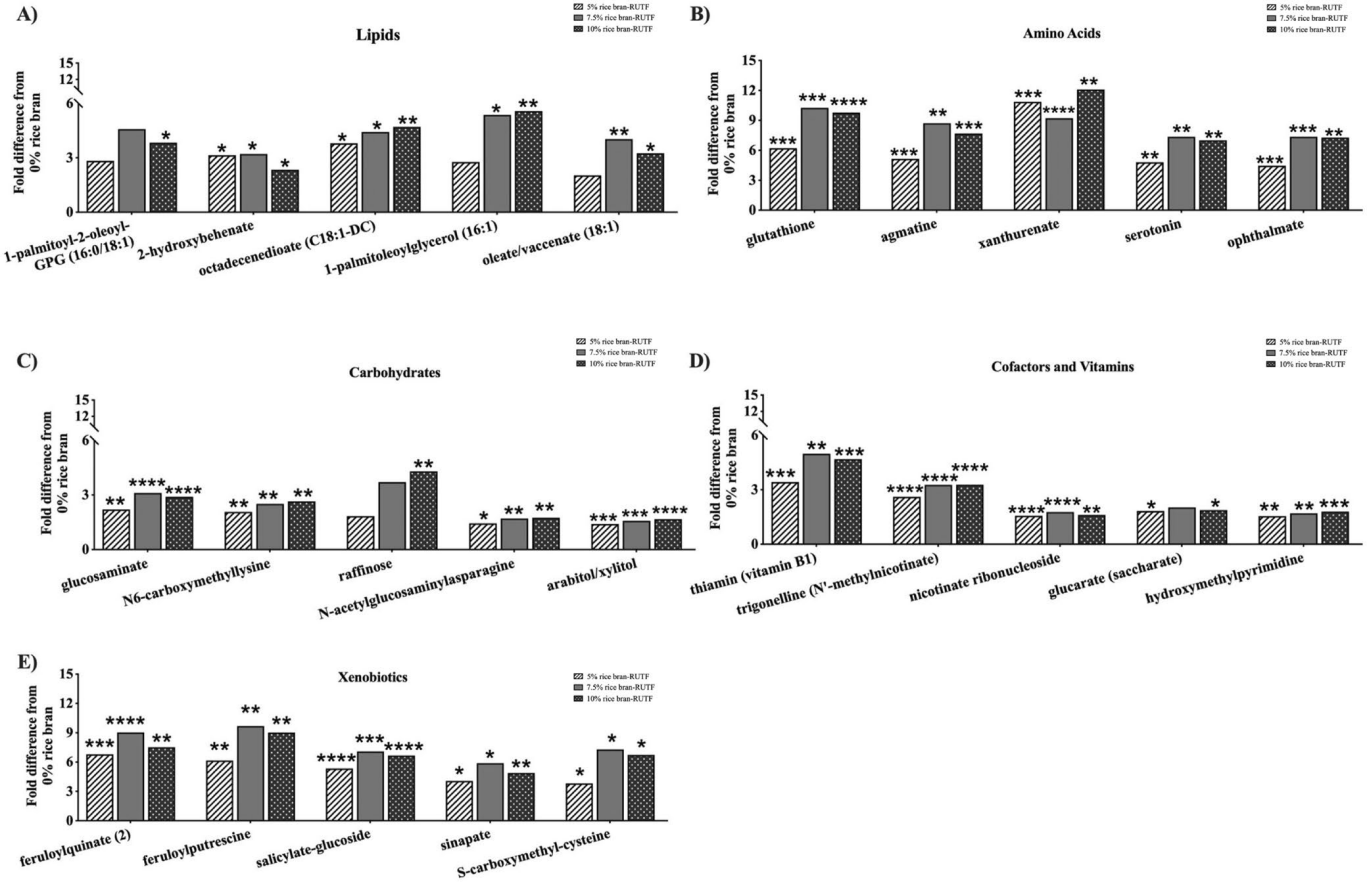
Select metabolite fold differences of log‐transformed median scaled relative abundance in comparison to 0% rice bran‐RUTF. Metabolites were selected based on top VIP scores for the chemical classes (A) Lipids, (B) Amino Acids, (C) Carbohydrates, (D) Cofactors and Vitamins, and (E) Xenobiotic compounds. Welch's *t*‐tests were used to determine statistical significance. The unadjusted *p*‐value is shown as *p ≤ 0.05; **p ≤ 0.01; ***p ≤ 0.001, ****p ≤ 0.0001.

Figure 2 illustrates five metabolites top‐ranked in VIP scores (Table S2) within the chemical classes Amino Acids, Carbohydrates, Cofactors and Vitamins, Lipids, and Xenobiotics and depicts fold differences of the rice bran‐RUTFs (5% rice bran‐RUTF, 7.5% rice bran‐RUTF, and 10% rice bran‐RUTF) compared to the RUTF without rice bran (0% rice bran‐RUTF). All metabolite fold differences, *p*‐values, and *q*‐values can be found in Table S3.

Within the Lipid chemical class, fatty acids related to health benefits were octadecenedioate and palmitoleoylglycerol. These all increased with the addition of rice bran (Figure 2A) and have been reported to provide health benefits such as cancer prevention (Ju et al. 2025) and neuroprotection properties (Lee et al. 2022). Metabolite analysis also highlighted a statistically significant fold difference of oleate in the rice bran‐RUTFs in 7.5% rice bran‐RUTF (4.03‐fold difference) and 10% rice bran‐RUTF (3.24‐fold difference) (Figure 2A and Table S3). A previous study found that rice bran consumption in moderately hypercholesterolemic children led to favorable changes to their blood lipid profile, with fatty acids such as oleate being prominent discriminatory metabolites (Li et al. 2018). This is particularly relevant in the management of children with acute malnutrition, as it provides a beneficial source of healthy fats, particularly within the context of preventing the increasingly prevalent issue of the double burden of malnutrition.

Within the Amino Acid class, glutathione significantly increased in the 5% rice bran‐RUTF, 7.5% rice bran‐RUTF, and 10% rice bran‐RUTF compared to the 0% rice bran‐RUTF (6.22‐fold difference, 10.27‐fold difference, and 9.79‐fold difference, respectively) (Figure 2B and Table S3). This non‐linear metabolite response of this potent antioxidant may reflect other phytochemical interactions between rice bran bioactives and the RUTF matrix, especially when considering that the product underwent heat processing such as extrusion and baking. The greater fold difference in the 7.5% rice bran‐RUTF of glutathione may indicate a threshold at which bioactive concentrations from rice bran help to stabilize relatively unstable compounds such as glutathione (Zhang et al. 1997). Such relationships are important to understand because glutathione, a potent antioxidant, plays major roles in preventing and controlling oxidative stress (Lengo et al. 2024). Our completed clinical trial in Mali showed that infants had significantly increased relative abundance of blood glutathione after 3 months of consuming rice bran compared to control (Pfluger et al. 2022). Also, oxidative stress and low glutathione have been linked to the development of edematous malnutrition (Di Giovanni et al. 2016; Golden and Ramdath 1987). Tryptophan Metabolism compounds such as xanthurenate and serotonin were also discriminatory factors related to the differences between the RUTFs in this non‐targeted metabolomics analysis. Xanthurenate has been documented as a candidate biomarker related to rice bran consumption in children (Baxter et al. 2022). Serotonin is an important neurotransmitter in cognitive function and derives from tryptophan metabolism (Jenkins et al. 2016), emphasizing rice bran influence to the gut‐brain axis. Children with malnutrition are typically low in plasma tryptophan, and enriched RUTFs to correct for these amino acid deficiencies are crucial (Sato et al. 2021). Good quality protein is crucial for malnutrition treatment, and the diverse amino acids profile demonstrated by this non‐targeted metabolite analysis demonstrates the added value of rice bran to these RUTFs.

Within the Carbohydrate chemical class, prebiotic soluble fiber metabolites such as raffinose increased with greater percentages of rice bran and had a statistically significant fold difference in the 10% rice bran‐RUTF (4.30‐fold difference) (Figure 2C Table S3). Importantly, raffinose has been found to support the immune system through the promotion of diverse probiotic bacteria and protection from enteric pathogens (Kanwal et al. 2023; Wongputtisin et al. 2015) and significantly increased in the 10% rice bran‐RUTF. Other carbohydrate metabolites such as arabitol/xylitol significantly increased in the 5% rice bran‐RUTF, 7.5% rice bran‐RUTF, and 10% rice bran‐RUTF compared to the 0% rice bran‐RUTF (1.41‐fold difference, 1.58‐fold difference, and 1.67‐fold difference, respectively) (Figure 2C and Table S3). Arabitol/xylitol have noted digestive benefits, playing a role in stimulating both the digestive and immune systems while having the added potential to reduce constipation, diabetes, and obesity (Gasmi Benahmed et al. 2020). This diverse fiber profile supplemented by rice bran would, therefore, be able to help supply substrates for commensal probiotic gut bacteria and promote a diverse gut microbiome.

Cofactors and Vitamins such as nicotinate ribonucleoside (NR), a vitamin B3 derivative, were major discriminatory factors in differentiating the rice bran‐RUTFs, and the fold difference was significant in 5% rice bran‐RUTF, 7.5% rice bran‐RUTF, and 10% rice bran‐RUTF compared to the 0% rice bran‐RUTF (1.57‐fold difference, 1.78‐fold difference, and 1.61‐fold difference, respectively) (Figure 2D and Table S3). NR is commonly used to boost levels of nicotinamide adenine dinucleotide (NAD+), reduce inflammation, and repair DNA (Hou et al. 2018). Another important metabolite that increased in relative abundance along with a greater percent of rice bran in the RUTFs was trigonelline, a methylation product of niacin. Trigonelline has been established as a biomarker of rice bran consumption (Baxter et al. 2022; Zarei et al. 2017), and is associated with various health benefits including antioxidant and anti‐inflammatory properties (Costa et al. 2020; Lorigooini et al. 2020) and the ability to improve muscle function (Membrez et al. 2024). Such attributes are particularly beneficial in malnutrition treatment as undernutrition is associated with heightened states of chronic inflammation.

Within the chemical class of Xenobiotics, many compounds responsible for distinctions between the different rice bran RUTF doses compared to no rice bran were phytochemicals such as ferulic acid metabolites (feruloylquinate and feruloylputrescine) and salicylate‐glucoside (Figure 2E). Feruloylquinate metabolites are bioactive phenolic compounds found in plants and are known for their role in preventing oxidative stress (Boulebd et al. 2023). Feruloquinate (2) was a major discriminatory metabolite differentiating the rice bran‐RUTFs and had a statistically significant fold change in 5% rice bran‐RUTF, 7.5% rice bran‐RUTF, and 10% rice bran‐RUTF compared to the 0% rice bran‐RUTF (6.80‐fold difference, 9.04‐fold difference, and 7.54‐fold difference, respectively) (Figure 2E and Table S3). Salicylate‐glucoside, a conjugate of salicylic acid, is a plant metabolite found in rice bran (Nealon et al. 2019) with well‐established health benefits including in managing inflammation and offering protection against certain diseases such as cardiovascular disease and cancer (Amann and Peskar 2002; Liu et al. 2023). In the rice bran‐RUTFs, there was a statistically significant increase in salicylate‐glucoside compared to the RUTF without rice bran (5.35‐fold difference in 5% rice bran‐RUTF, 7.10‐fold difference in 7.5% rice bran‐RUTF, and 6.68‐fold difference in 10% rice bran‐RUTF) (Figure 2E and Table S3). The increase in bioactive rice bran‐derived metabolites suggests the added functional food ingredient value of rice bran in RUTFs for malnutrition treatment alongside child growth and development of the gut‐brain axis. Overall, the non‐targeted metabolomic analysis revealed that greater rice bran inclusion rates markedly enhanced the metabolite composition, supporting the improved functional and nutritional quality of the RUTFs.

### 3.3 Targeted Nutrient Analysis of Small‐Scale Mill‐Sourced Rice Brans

To better understand the application and potential for in‐country incorporation of rice bran in malnutrition treatment, small‐scale mill‐sourced rice brans from countries with high levels of malnutrition and where rice is grown were analyzed. Rice bran collected from small‐scale mills in Guatemala and Cambodia was analyzed and compared to commercial‐US rice bran (SN100 rice bran, Stabil Nutrition, St. Louis, MO, USA), the same rice bran used in the RUTFs previously described. Important in the interpretation of the results is the limitation that the nutrient composition measurements were conducted in single replicates (*n* = 1) for each rice bran sample. Table 3 illustrates the differences in vitamin content of multiple global rice bran varieties. Of the mill‐sourced rice brans, Guatemala II and Cambodia‐Jasmine Dry Season Mill II contained relatively high levels of various B vitamins and vitamin E. However, overall vitamin content differed greatly by region and mill.

**TABLE 3:** Quantified vitamin content of small‐scale mill‐sourced rice bran from Guatemala and Cambodia compared to a US‐commercial rice bran.

Vitamin (mg/100 g)	Rice Bran Variety								
	Guatemala I	Guatemala II	Cambodia Jasmine Wet Season Mill I	Cambodia Jasmine Dry Season Mill I	Cambodia Non‐Jasmine Mill I	Cambodia Jasmine Wet Season Mill II	Cambodia Jasmine Dry Season Mill II	Cambodia Non‐Jasmine Mill II	US SN100
Vitamin B1	< 0.1	< 0.1	0.19	0.19	< 0.1	< 0.005	0.034	0.012	2.35
Vitamin B2	0.27	0.28	0.35	0	0	0.06	0.06	0.05	0.44
Vitamin B3	20.8	37.2	35	34.6	36.1	40.79	41.52	40.97	46.8
Vitamin B5	5.1	7.9	7	7	8	0.01	0.018	0.015	1.93
Vitamin B6	1.9	3.2	3.8	3.6	4.4	< 0.005	< 0.005	< 0.005	0.54
Vitamin B9 (μg/100 g)	45	70	72	66	70	< 1	< 1	< 1	98.2
Vitamin E	3.2	5.2	4.8	5	6	4.8	5.6	5.0	5.1

Vitamin B1 was relatively low in the mill‐sourced rice brans compared to the commercial‐US rice bran, indicating that vitamin B1‐rich rice bran depends on the region grown. Vitamin B1 deficiencies are prevalent around the world, especially in Southeast Asia (Barennes et al. 2015; Whitfield et al. 2017), where diets rely largely on white rice which lacks adequate vitamin B1. While rice bran certainly contributes to higher intakes, additional vitamin B1‐rich ingredients, such as legumes and nuts, should be included in the diet to address dietary deficiencies.

All tested rice brans were rich in vitamin B3, averaging 37.1 mg/100 g across the rice bran varieties. Vitamin B3 was also high in mill‐sourced rice brans in Guatemala and Cambodia, providing a rationale for locally sourced rice brans to enhance a nutrient‐dense diet. The Guatemalan rice bran I and II, as well as rice bran from Cambodia Mill I (Jasmine Wet Season, Jasmine Dry Season, Non‐Jasmine), were rich in vitamin B5 and B6, and were greater than the commercial‐US rice bran. Rice bran from Cambodia Mill II (Jasmine Wet Season, Jasmine Dry Season, Non‐Jasmine) was relatively low in vitamin B6 (< 0.005 mg/100 g). All tested rice bran from Guatemala and Cambodia had vitamin E concentrations comparable with the commercial‐US rice bran (3.2–6.0 mg/100 g). These variations highlight the difference in rice bran by geographical location where the rice was grown.

Table 4 presents the microbiological and heavy metal testing of these rice brans to provide insights for the critical food safety analysis of these globally sourced rice brans. All rice brans tested negative for *Salmonella* spp. The Cambodia‐Non‐Jasmine I and Cambodia‐Jasmine Wet Season Mill I both had the highest levels of *E. coli* (≥ 600 CFU/g), and yeast and mold were highest in the Cambodia‐Jasmine Wet Season Mill I and Cambodia‐Jasmine Dry Season Mill I (≥ 210,000 CFU/g). Such results indicate possible contamination possibly due to the wet season conditions. The SN100 had the lowest levels of all microbiological tests, exemplifying the monitoring and food safety checks that a commercial product undergoes, and emphasizes the importance of this testing in other small‐scale mills for optimal food safety.

**TABLE 4:** Microbiological evaluation and heavy metal concentrations of small‐scale mill‐sourced rice bran from Guatemala and Cambodia compared to a US‐commercial rice bran.

	Rice Bran Variety					
	Guatemala I	Guatemala II	Cambodia Jasmine Wet Season Mill I	Cambodia Jasmine Dry Season Mill I	Cambodia Non‐Jasmine Mill I	US SN100
Microbiological (CFU/g)						
*Salmonella* spp.	Neg	Neg	Neg	Neg	Neg	Neg
*E. coli*	80	20	600	< 10	3700	< 10
Yeast and Mold count	8200	133	240,000	210,000	73,000	100
Total enteric count	340,000	11,000	73,000	45,000	78,000	10,000
Heavy Metals (mg/kg)						
Lead	0.09	0.16	0.06	0.02	0.07	0.02
Cadmium	0.06	0.08	< 0.01	< 0.01	< 0.01	0.02
Arsenic	0.04	0.06	0.66	0.77	0.79	0.76

It is recommended to add a post‐harvest and post‐milling step for rice bran in supply chains from regions such as Guatemala and Cambodia. The additional steps for proper storage techniques that use well‐ventilated spaces and controlled temperature and humidity (Gonçalves et al. 2019) are critical with implementation of regular monitoring of microbial counts. In addition, immediate heat stabilization of rice bran after milling is essential to inactivate endogenous lipase enzymes to reduce rancidity and prevent microbial growth (Das et al. 2024). Techniques such as dry heat treatment, extrusion, microwave heating, or infrared stabilization can effectively lower both lipase activity and microbial contamination, thereby improving the shelf life and safety of rice bran (Das et al. 2024). Further processing treatments such as using cold plasma, pulsed light, ozonation, microwave, irradiation and plant antifungal metabolites addition are also effective in reducing mycotoxin loads in rice bran (Chandravarnan et al. 2022; Oliveira et al. 2012).

Levels of lead and cadmium were lowest in Cambodian rice brans and in the SN100, and arsenic was the lowest in the Guatemalan rice brans (≤ 0.06 mg/kg) (Table 4). Such findings demonstrate the large variability that exists for trace metal contamination by region, and as previously demonstrated (Ngo et al. 2024; Sedeek et al. 2023; Weber et al. 2021). For local rice bran incorporation into food systems and supply chains, it is critical that regulations in screening are performed to understand vitamin/mineral content, as well as to monitor for food safety concerns.

This analysis of globally sourced rice brans revealed variability in vitamin composition, microbiological tests, and heavy metal concentrations, which highlights the importance for standardized nutrient screening and food safety control measures prior to the incorporation into malnutrition treatment foods.

### 3.4 Global Application of Rice Bran in Malnutrition Treatment Foods

The first use of rice bran in a locally produced RUTFs for gut microbiome‐targeted malnutrition treatment was tested in the SEHAT randomized controlled trial in Jember, Indonesia (Weber et al. 2023) (NCT05319717). In the SEHAT clinical trial, children received RUTF formulations containing either 0% or 5% rice bran to evaluate the contribution of rice bran in the treatment of malnutrition. The RUTFs analyzed in the present study closely reflect those used in the SEHAT trial but were prepared without the added vitamin/mineral premix to isolate specific nutrient contributions from rice bran. Notably, the 5% rice bran–RUTF demonstrated a > 2‐fold increase in dietary fiber and ~2‐fold higher vitamin B1 concentration compared to 0% RUTF. Non‐targeted metabolomic profiling further identified enrichment of various metabolites, including elevated amino acids, lipids, and bioactive phytochemicals such as feruloylquinates and salicylate‐glucosides. These biochemical improvements highlight the potential of rice bran to enhance the nutrient density of RUTFs. The compositional differences underscore the nutritional advantages of rice bran inclusion and support its continued evaluation in regionally produced malnutrition treatment foods. Given Indonesia's status as a major rice producer (Siahaan 2024) and the high malnutrition rates (Indonesian Ministry of Health 2023), introducing rice bran into locally produced RUTFs offers significant potential for addressing malnutrition both in Indonesia and other regions.

There is a growing need to improve functional food product formulations to meet cultural preferences, and to provide affordable, locally sourced options. Guatemalan and Cambodian rice brans tested from local rice milling operations present promising opportunities for utility in malnutrition treatment foods and food‐nutritional security programs. In Guatemala, nearly half of children under five are affected by chronic malnutrition (USAID 2021). Cambodia also struggles nationally with high levels of stunting and micronutrient deficiencies, contributing to poor health outcomes over the lifespan (Whitfield et al. 2017). Rice bran collected from small‐scale Guatemalan mills contained elevated levels of many B vitamins, and trace metal concentrations were low, indicating strong potential for safe, local use of rice bran. Rice bran from Cambodian small‐scale mills, particularly Jasmine Dry and Wet Season mills, was also rich in B vitamins as well as vitamin E. However, microbiological testing demonstrated elevated yeast and mold counts in wet season samples, and underscores the need for improved stabilization and moisture control during storage. With proper post‐harvest handling, Cambodian rice bran could provide a local nutrient‐rich and cost‐effective ingredient. Understanding the nutrient density of rice bran from these countries provides key insights into locally sourced ingredients that can contribute to therapeutic food formulations and are more affordable than imported alternatives.

These results support the use of rice bran as a partial substitution for vitamin/mineral premix as previously examined in Kenya (Kinyuru et al. 2015). It is important to note, however, that alternative ingredients, such as rice bran, can act only as a complement or partial substitute for an RUTF premix of vitamins and minerals, as these commercial blends are specifically formulated to allow recovery and catch‐up from severe acute malnutrition, which requires higher daily intakes (Golden 2009). A new wave of RUTF product development across the globe has been pushing for enhancing malnutrition treatment efficacy and long‐term health disparities using local and culturally relevant ingredients.

Utilizing locally produced rice bran supports sustainability goals, as its production requires minimal additional energy or land use. Domestic sourcing of rice bran can also reduce reliance on imported ingredients, lower transportation costs and emissions, as well as strengthen regional food systems and local economies. As rice bran is produced in large quantities worldwide, it offers a widely available and cost‐effective ingredient for RUTF formulations. However, scaling up its use will require investment from local governments and industry partners to build quality control systems and ensure product consistency and safety. Existing small‐scale rice mills in countries such as Guatemala and Cambodia could be integrated into RUTF production operations and could enable sustainable, locally produced malnutrition treatment foods. Overall, these findings highlight promising opportunities for continued research and product innovation.

### 3.5 Study Limitations

This study offers important insights into how rice bran affects RUTF nutrient density; however, future clinical trials assessing growth, gut health, and recovery outcomes are needed to determine the functional benefits of rice bran in malnutrition treatments. A key limitation in the targeted nutrient analysis was the single measurement (*n* = 1) testing of the RUTFs and rice bran samples. Additional analyses would be valuable to confirm nutrient levels. For example, the 10% rice bran‐RUTF met Codex guidelines for vitamin A, though rice bran itself is not typically a rich source of this vitamin. The RUTF targeted nutrient results, however, are based on representative batch samples prepared from 30 homogenized wafer rolls (15 chocolate and 15 vanilla), which provides a reasonable reflection of the formulation despite being a single measurement. Furthermore, while nutrient composition was assessed, this study did not evaluate the bioaccessibility or bioavailability of these nutrients, which is directly related to their physiological impact. Research should continue to focus on clinical trials to evaluate the efficacy of rice bran‐based food product formulations in real‐world settings. Such studies provide evidence for the local production of RUTFs and can help inform policies related to malnutrition treatment programs.

## 4 Conclusion

Rice bran inclusion in RUTFs increased nutrient density and enhanced key bioactive compounds, including amino acids, lipids, and bioactive phytochemicals. These biochemical enrichments demonstrate the potential of rice bran to improve the nutritional and functional quality of malnutrition treatment foods. Findings from the targeted nutrient analysis indicate Codex guidelines for vitamin B3 and E can be met prior to the addition of a vitamin/mineral premix. Collectively, these findings support rice bran as a valuable, locally sourced ingredient that can strengthen RUTF formulations and contribute to sustainable strategies for advancing global food and nutritional security.

## Author Contributions

**Annika M. Weber:** data curation (lead), formal analysis (lead), investigation (lead), methodology (lead), visualization (lead), writing – original draft (lead), writing – review and editing (lead). **Emma S. Bovaird:** data curation (equal), formal analysis (equal), investigation (equal), methodology (equal), writing – original draft (equal), writing – review and editing (equal). **Sahar B. Toulabi:** formal analysis (equal), methodology (equal), visualization (equal), writing – review and editing (equal). **Silvia Barbazza:** writing – review and editing (equal). **Moretta Damayanti Fauzi:** writing – review and editing (equal). **Fildzah K. Putri:** methodology (equal), writing – review and editing (equal). **Khaerul Fadly:** data curation (equal), methodology (equal), writing – review and editing (equal). **Kharisma Tamimi:** data curation (equal), methodology (equal), writing – review and editing (equal). **Diva M. Calvimontes:** writing – review and editing (equal). **Rimbawan Rimbawan:** data curation (lead), methodology (lead), writing – review and editing (equal). **Zuraidah Nasution:** data curation (lead), methodology (lead), writing – review and editing (equal). **Puspo Edi Giriwono:** conceptualization (equal), data curation (lead), investigation (lead), project administration (equal), writing – review and editing (equal). **Frank T. Wieringa:** conceptualization (lead), funding acquisition (lead), methodology (lead), project administration (lead), supervision (equal), writing – review and editing (equal). **Elizabeth P. Ryan:** conceptualization (lead), funding acquisition (lead), methodology (lead), project administration (lead), supervision (lead), writing – review and editing (equal).

## Funding

This work was supported by the Thrasher Research Fund, and by the National Institute of Child Health and Human Development of the National Institutes of Health under Award Number 1R21HD113211 (PI: Ryan). A.M.W. was supported by the National Institute of Allergy and Infectious Diseases of the National Institutes of Health under Award Number T32AI162691. The research presented in this manuscript was conducted as part of the PhD dissertation for A.M.W. at Colorado State University (EPR thesis advisor) and was completed prior to them performing consultant status with Stabil Nutrition.

## Conflicts of Interest

The authors declare no conflicts of interest.

## Supporting information


**Figure S1:** Methods workflow for experimental RUTF nutrient analysis. *Conducted separately for 0%, 5%, 7.5%, and 10% rice bran‐RUTFs samples. The figure was created with BioRender.com.

**Table S1:** Nutritional analysis of SN100, Stabil Nutrition*

**Table S2:** Metabolites with variable importance in projection (VIP) scores > 1.0 identified from partial least squares–discriminant analysis (PLS‐DA). VIP score reflects a measure of metabolite impact as a discriminant feature among the RUTFs.

**Table S3:** Fold differences in metabolite median scaled relative abundances for 5%, 7.5%, and 10% rice bran–RUTF compared to 0% rice bran–RUTF. Statistical significance was assessed using Welch's two‐sample *t*‐test with corresponding *p*‐values and *q*‐values to account for multiple comparisons.

## Data Availability

Data not already provided in the Supporting Information will be made available upon request to the corresponding author.
