# Supplementary material for: Rice Bran Supplements the Nutritional Density of Ready‐to‐Use Therapeutic Foods: A Targeted Nutrient and Non‐Targeted Metabolomic Analysis
Source: Food Sci Nutr. 2026 Jan 21;14(1):e71448. doi: 10.1002/fsn3.71448 (PMC12824455; doi:10.1002/fsn3.71448)
Supplement: Supplementary file 1 — Figure S1: Methods workflow for experimental RUTF nutrient analysis. *Conducted separately for 0%, 5%, 7.5%, and 10% rice bran‐RUTFs samples. The figure was created with BioRender.com. [file FSN3-14-e71448-s001.pptx]

## Slide 1
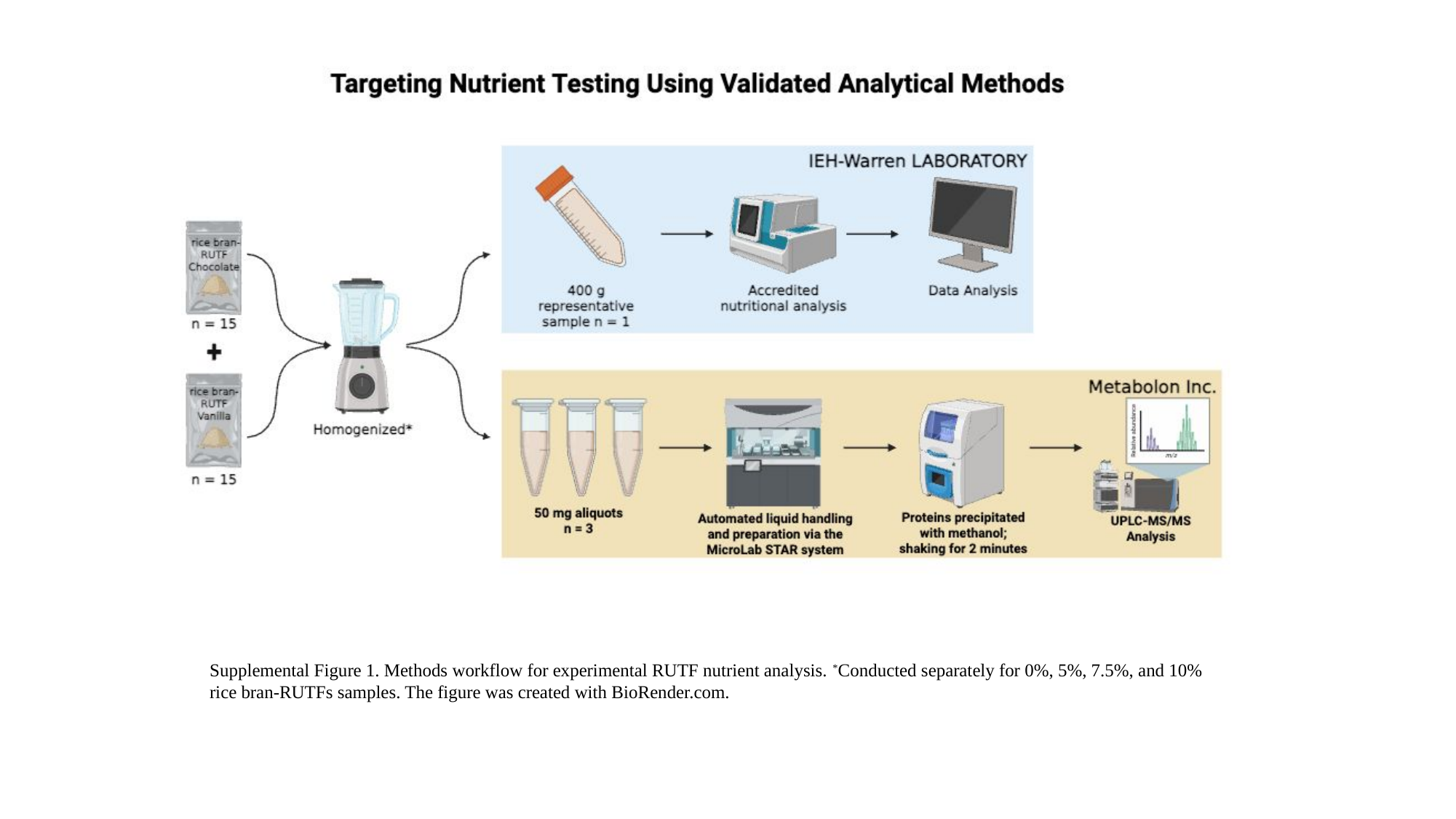

Supplemental Figure 1. Methods workflow for experimental RUTF nutrient analysis. *Conducted separately for 0%, 5%, 7.5%, and 10% rice bran-RUTFs samples. The figure was created with BioRender.com.
