# Supplementary material for: Rice Bran Supplements the Nutritional Density of Ready‐to‐Use Therapeutic Foods: A Targeted Nutrient and Non‐Targeted Metabolomic Analysis
Source: Food Sci Nutr. 2026 Jan 21;14(1):e71448. doi: 10.1002/fsn3.71448 (PMC12824455; doi:10.1002/fsn3.71448)
Supplement: Supplementary file 2 — Table S1: Nutritional analysis of SN100, Stabil Nutrition* [file FSN3-14-e71448-s004.docx]

| **Supplemental Table 1.** Nutritional analysis of SN100, Stabil Nutrition^*^ | | | | | | |
| --- | --- | --- | --- | --- | --- | --- |
| ***Nutrient*** | ***Unit*** | | ***per 100 g*** | | | ***Method*** |
| **PROXIMATES** | | | | | | |
| Protein | g | | 16.12 | | | AOAC 991.20.1 |
| Total Fat | g | | 19.52 | | | AOAC 960.39 |
| Total Carbohydrate | g | | 49.20 | | | Calculation |
| Dietary Fiber | g | | 21.57 | | | AOAC 991.43 |
| Soluble Fiber | g | | 4.48 | | | AOAC 991.43 |
| Insoluble Fiber | g | | 17.09 | | | AOAC 991.43 |
| Ash | g | | 8.45 | | | AOAC 925.51A |
| Moisture | g | | 6.68 | | | ASTA 2.1 |
| Calories | kcal | | 360 | | | Calculation |
| **VITAMINS** | | | | | | |
| Thiamin, B1 | | mg | | 2.35 | AOAC 942.23 | |
| Riboflavin, B2 | | mg | | 0.44 | AOAC 970.65 | |
| Niacin, B3 | | mg | | 46.8 | AOAC 944.13 | |
| Pantothenic acid, B5 | | mg | | 1.93 | AOAC 960.46 & Kit | |
| Pyridoxin, B6 | | mg | | 0.54 | AOAC 960.46 & Kit | |
| Folate, B9 | | mcg | | 98.2 | AOAC 960.46 & Kit | |
| Vitamin E | | mg | | 5.1 | AOAC 992.03 | |
| ^*^Analysis completed by: Merieux NutraSciences/Silliker, Inc, 3600 Eagle Nest Dr, Crete, IL 60417 for Stabil Nutrition, March 2020. | | | | | | |
